# A two-year follow-up: Twitter activity regarding misinformation about spinal manipulation, chiropractic care and boosting immunity during the COVID-19 pandemic

**DOI:** 10.1186/s12998-022-00469-7

**Published:** 2023-01-23

**Authors:** Gregory Neil Kawchuk, Steen Harsted, Jan Hartvigsen, Luana Nyirö, Casper Glissmann Nim

**Affiliations:** 1 Department of Physical Therapy, University of Alberta, Edmonton, Canada; 2 Department of Sports Science and Clinical Biomechanics, University of Southern Denmark, Odense, Denmark; 3 Chiropractic Knowledge Hub, Odense, Denmark; 4 Department of Chiropractic Medicine, Balgrist University Hospital, University of Zurich, Zurich, Switzerland; 5 Medical Research Unit, Spine Centre of Southern Denmark, University Hospital of Southern Denmark, Middelfart, Denmark; 6 Department of Regional Health Research, University of Southern Denmark, Odense, Denmark

**Keywords:** Social media, Twitter, Spinal manipulation, Chiropractic, Misinformation, Immunity

## Abstract

**Background:**

Spinal manipulative therapy (SMT) is offered by many health professions, most often by chiropractors. While SMT can be effective for some musculoskeletal disorders, there is no evidence that SMT improves human immunity in a clinically meaningful way. Despite this, we showed previously that Twitter misinformation about chiropractic/SMT  improving immunity increased sharply at the start of the COVID-19 pandemic. Here, we perform a two-year follow-up.

**Methods:**

We previously employed specialized software (i.e. Talkwalker) to search the entirety of Twitter activity in the  months before and after the COVID-19 pandemic was declared (March 11, 2020). In this paper, we conducted follow-up searches over two successive 12 month periods using terms related to SMT, immunity and chiropractic. The resulting tweets were then coded into those promoting/refuting a relation between SMT and immunity (tone) and messaging about chiropractic/interventions (content). Further analyses were performed to subcategorize tweet content, tally likes, retweets and followers, and evaluate refuting tweets and the country of origin. Finally, we created a chronology of Twitter activity superimposed with dates of promoting or refuting activities undertaken by chiropractic organizations.

**Results:**

Over the 27 month study period, Twitter activity peaked on March 31, 2020 then declined continuously. As in our first paper, our follow-up data showed that (1) the ratio of refuting/promoting tweets remained constant and (2) tweets that refuted a relationship between SMT and immunity were substantially more liked, retweeted and followed than those promoting. We also observed that promoting tweets suggesting that SMT improves immunity decreased more rapidly. Overwhelmingly, promoting tweets originated in the USA while refuting tweets originated in Canada, Europe and Australia. The timing of the decline in peak Twitter activity, together with a parallel decline in tweets claiming that SMT improves immunity, was coincident with initiatives by chiropractic organizations and regulators targeting misinformation.

**Conclusion:**

Overwhelmingly, Twitter activity during the COVID-19 pandemic focussed on refuting a relation between chiropractic/SMT and immunity. A decline in Twitter activity promoting a relation between SMT and immunity was observed to coincide with initiatives from chiropractic organizations and regulators to refute these claims. The majority of misinformation about this topic is generated in the United States.

## Introduction

On March 11, 2020, the World Health Organization officially declared a pandemic for COVID-19 [1]. Since then, the SARS-CoV-2 virus has been responsible for more than 550 million infections and 6 million deaths [2].

As the pandemic evolved, the resulting mass of information generated by media, government, scientists and social media created what has become known as an “infodemic” [3]. Navigating this infodemic has become a challenge not only because of its sheer volume, but also because of the patchwork nature of information describing regional policies, spot outbreaks, new variants, and research distribution.

Adding to this challenge was the emergence and growth of pandemic-specific misinformation [4]. Defined as inaccurate information spread without specific intent, misinformation occurs regularly in daily life such as a misquoted address, but on a global level, misinformation wreaked havoc on efforts to combat the pandemic; sometimes with fatal consequences [4]. Specifically, the WHO has stated, “The unfolding of the COVID-19 pandemic has demonstrated how the spread of misinformation, amplified on social media and other digital platforms, is proving to be as much a threat to global public health as the virus itself [5].”

As a result, studying the genesis, spread and evolution of pandemic misinformation is a growing academic area that now places misinformation into categories about the virus itself, vaccines, politics, conspiracy theories and possible cures and/or interventions [6]. Early in the pandemic, before vaccinations or anti-viral drugs were available, a great amount of misinformation was focused on interventions purported to “boost” immunity for the prevention or mitigation of covid infections [7]. These “boosting” interventions are typically associated with optimizing basic human functions such as eating, sleep, exercise, or through specific products like nutritional supplements.

Included in these “immune-boosting” interventions is Spinal Manipulative Therapy (SMT) [8]. Most commonly delivered by chiropractors in the management of musculoskeletal conditions, SMT is sometimes promoted as having systemic effects including the ability to boost immunity [9]. While there is evidence supporting the use of SMT as an intervention for low back pain and other MSK conditions [10], we are not aware of any robust evidence that SMT, nor a specific profession that provides SMT, creates a clinically meaningful improvement in the human immune system [11, 12].

Nonetheless, we have shown that social media claims of a positive association between SMT provided in a chiropractic context, and boosting immunity, rose sharply at the onset of the pandemic [8]. Although these claims have also been documented outside of social media [9], our prior analysis of the entirety of Twitter data demonstrated that:

- Twitter misinformation claiming a positive relation between SMT and immunity increased dramatically during the onset of the COVID crisis compared to the 12 months prior.
- The potential reach (audience) of tweets refuting a link between SMT and immunity was 3 times higher than those promoting a link.
- Users with the greatest influence on Twitter, as either promoters or refuters, were individuals, not institutions or organizations.
- Of tweets mentioning a profession, chiropractic was most frequent.
- The majority of tweets promoting a relation between SMT and immunity were generated in the USA while the majority of refuting tweets originated from Canada.

Since then, we have collected two years of follow-up data with the goal of determining if, and how, Twitter messaging regarding SMT and immunity has evolved during the pandemic. Here, we compare Twitter data from the first 3 months of the pandemic (January 2020–March 2020), the next 12 months of the pandemic (April 2020–April 2021) and then the following 12 months (April 2021–April 2022).

Given the number of efforts by the chiropractic profession aimed at decreasing misinformation about chiropractic/SMT and immunity during the early pandemic, we hypothesized that: (1) tweet frequency regarding chiropractic/SMT and immunity would decrease, (2) the proportion of refuting versus promoting tweets would remain stable over time and (3) the content (chiropractic/intervention) of the promoting tweets would change over time.

## Methods

### Search strategy

Social media searching was performed using Talkwalker Quick Search (Luxembourg, Luxembourg), the details of which we have published previously [8]. Talkwalker searches were performed exclusively on Twitter data for three time periods: period A (January 1, 2020–March 31, 2020), period B (April 1, 2020–March 31, 2021) and period C (April 1, 2021–March 31, 2022). We constructed our searches to identify tweets related to SMT, chiropractic and immunity. For period A (performed previously), our search terms were (adjust* OR manipulat* OR smt) AND (chiro* OR physio* OR “physical therap*” OR naturo* OR osteo* OR napra*) AND (immun*). Based on our prior results from this search that showed chiropractic to be the profession most often associated with SMT and claims of boosting immunity, search terms for periods B and C were constructed as (smt AND immun*) OR (chiro* AND immun*) AND NOT (immunocompromised) AND NOT (immune-compromised). The above searches identified tweets that contained the search terms in the body of the tweet as words and/or hashtags (e.g. #chiropractic). For each search result, individual tweet attributes were obtained including date, creator, messaging, country of origin, language, likes, retweets and followers.

### Coding of tweets

Resulting tweets were coded manually for their tone using the Twitter Tone Index (TTI). The TTI [8] is a nominal index of four coding options: (1) promoting a relation between SMT and/or a profession providing SMT and improved immunity, (2) refuting that same relation, (3) neutral messaging or (4) irrelevant messaging. Prior calibration resulted in a Fleiss Kappa score of 0.85 interpreted as almost perfect agreement [13]. Three evaluators (LN, SH, CN) independently assessed each tweet using the TTI. Tweets not having complete agreement were discussed until agreement was obtained. Tweets in all four categories were tallied. Only tweets that were promoting or refuting were taken forward for analysis.

Search results were then coded for mentions of professions/interventions by the same evaluators. First, tweets were coded using any combination of the following 5 categories: chiropractic mentioned, SMT mentioned, health advice mentioned (not chiropractic or SMT), supplements mentioned, or other interventions mentioned. Again, Tweets not having complete agreement were discussed to determine a majority rating. From these results, the 5 content categories create 120 possible combinations (5 factorial). These were then pooled into three main categories based on their content: Chiropractic care only (CC), SMT only (SMT) and Chiropractic care with non-SMT interventions (noSMT).

Engagement was defined as the likes plus retweets linked to any one tweet while reach was defined as the number of followers associated with a tweet.

### Data analysis

First, the number of tweets was tallied, as was engagement and reach, then stratified into promoting and refuting tweets. The data were then plotted over time as weekly totals and also plotted to show the proportion of promoting and refuting tweets for periods A, B and C.

We then divided absolute counts by the number of months in each period to arrive at monthly rates for tweets, measures of engagement and reach. These results were then plotted by period.

For refuting tweets, we tallied their content (chiropractic/intervention) coded as CC, SMT and CC noSMT for periods A, B and C then determined the percentage distribution of these three codes in each period. We then wanted to know if the percentage distribution of the three content codes in period A was preserved in period B and period C in order to determine if any change from period to period was spread equally between the three content codes, or if the content codes shifted unequally. We first divided code counts by the months in each period to determine the monthly rate of tweets for each code, then we did the same for the total number of codes in each period. Expecting the percentage distribution of the three content codes in period A would remain the same in period B, we calculated the expected change rate by dividing the total monthly rate in period B by the same in period A (39%) and then did the same from period B to C (46%). We then calculated the difference between the expected rate of change and the actual rate of change for each code in each period. This difference allowed us to determine if the changes in content codes distribution from period to period was spread equally across the three content codes, or if the codes changed unequally from period to period.

Tweets were then plotted geospatially with their individual latitude and longitude coordinates.

### Chronological event plot

In order to illustrate potential impact of activity from chiropractic organizations and regulators designed to combat misinformation about SMT/chiropractic and immunity, we plotted weekly tweet counts together with the dates of these activities. Specifically, between March 10 and March 31, 2020 several chiropractic organizations made formal announcements that emphasized the lack of evidence for chiropractic/SMT and a clinically significant improvement in human immunity. These announcements came from several sources including a joint announcement from organizations in the United Kingdom (British Chiropractic Association, McTimoney Chiropractic, Scottish Chiropractic Association, United Chiropractic Association, Royal College of Chiropractors) [14], the Canadian Chiropractic Association [15], the World Federation of Chiropractic [11], the European Union of Chiropractors Associate Members [16], a Facebook interview between the President of Parker University and a staff researcher [17], the American Chiropractic Association [18] and the Swedish Chiropractic Association [19].

In addition, several chiropractic regulators made official statements outlining the consequences of making misleading claims about chiropractic care, SMT, immunity and COVID infections (Fig. 6). Examples include statements from The College of Chiropractors of Alberta [20], The College of Chiropractors of British Columbia [21] and the Australian Health Practitioner Regulation Authority [22] with regulators from British Columbia [23] and Alberta using specialized software to monitor their member's social media activity.

Finally, we plotted announcements from chiropractic organizations and individuals with contrary messaging [24–27].

## Results

**Fig. 1:**
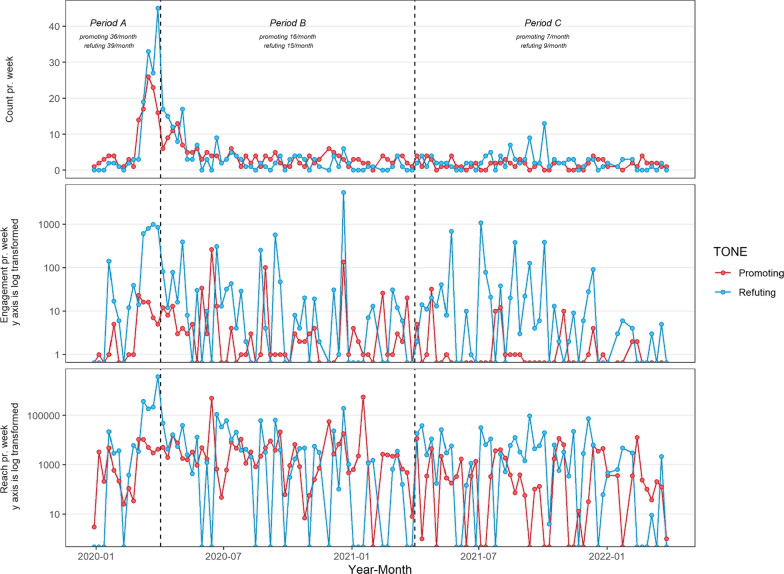
Weekly count of tweets, engagement (likes + retweets) and reach (followers)

**Fig. 2:**
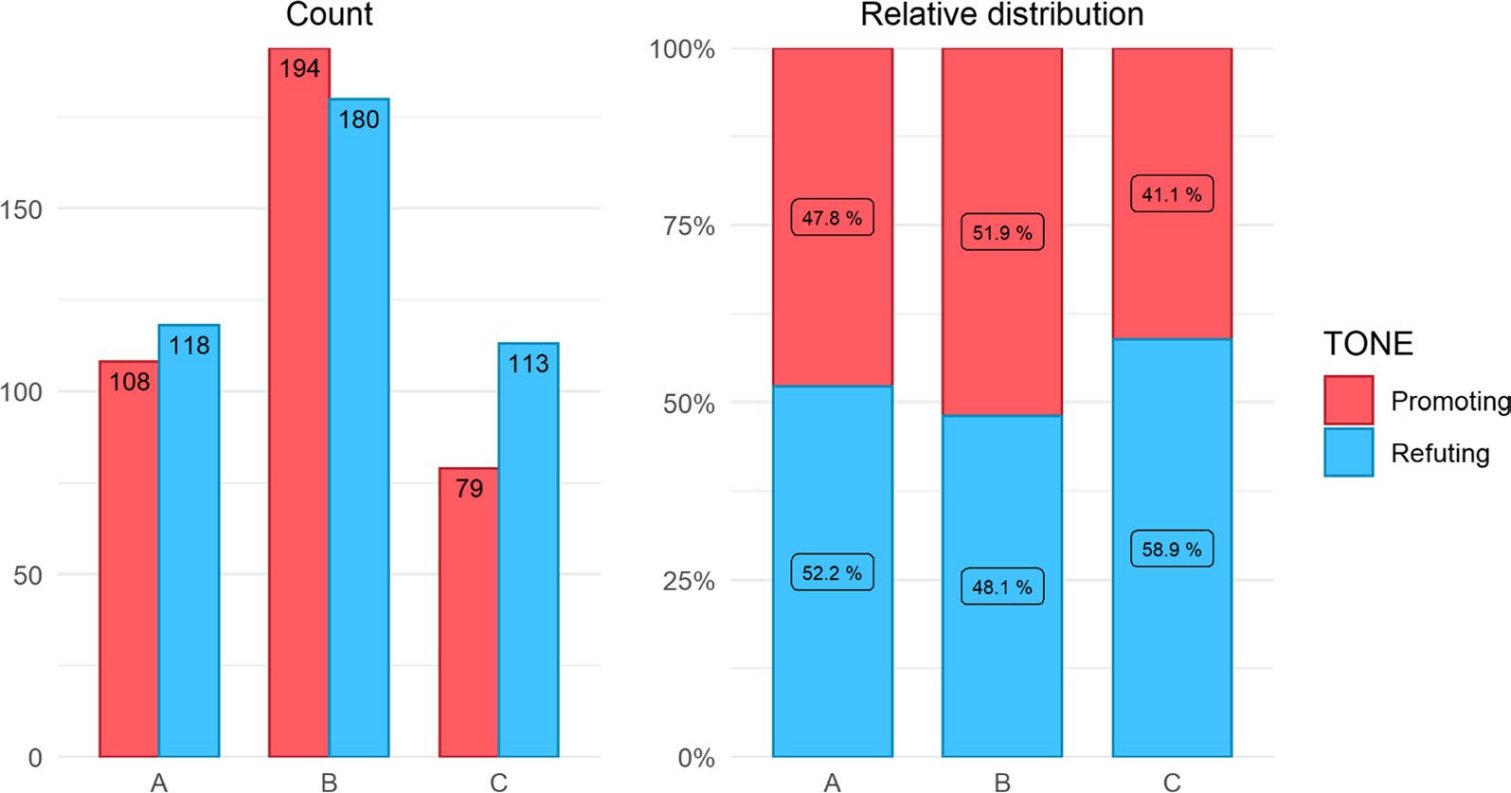
Absolute and relative distribution of promoting and refuting tweets by period

**Fig. 3:**
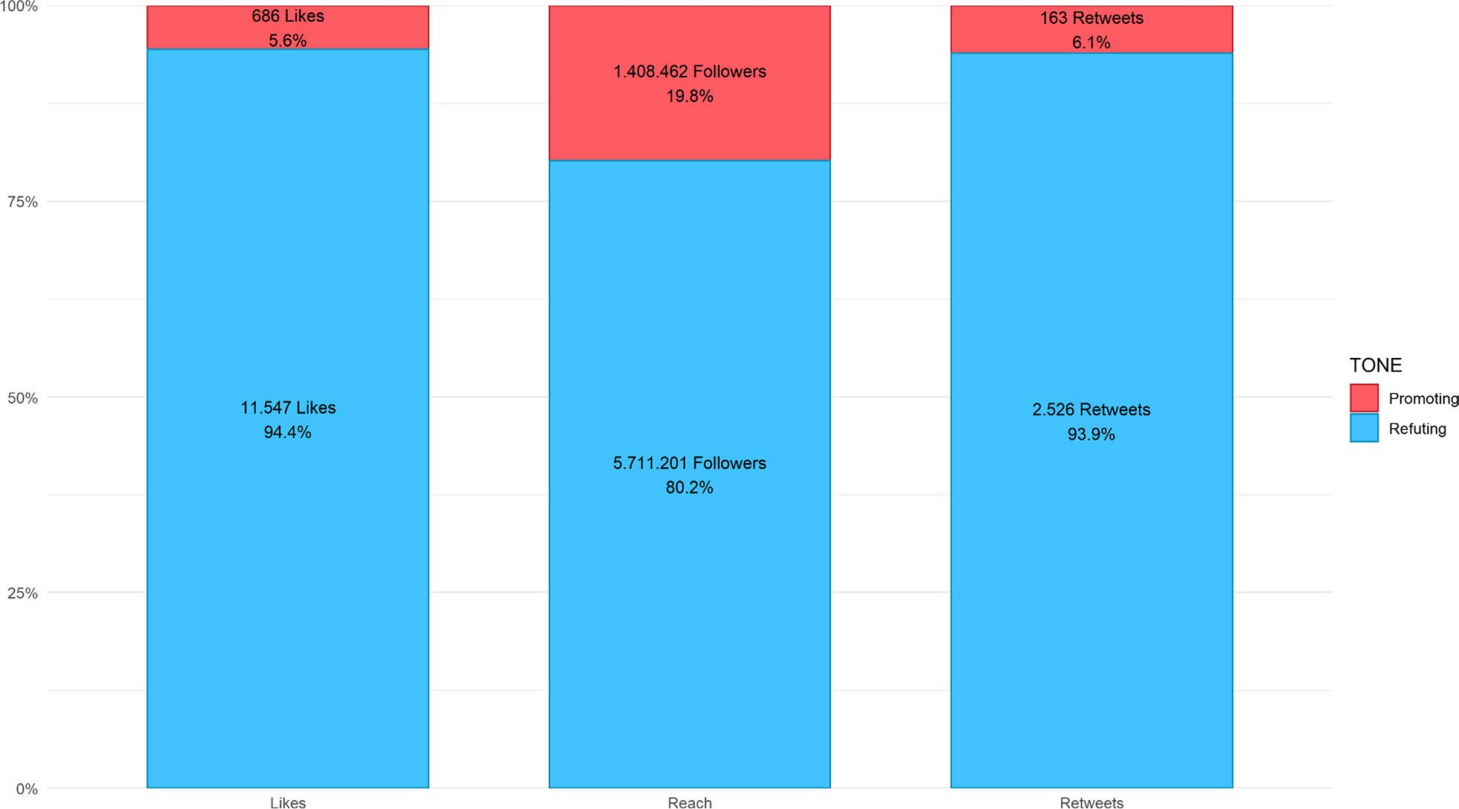
Total engagement (likes plus retweets) and reach (followers)

Our searches identified 916 tweets in total. After eliminating tweets coded as neutral (e.g. *“#ChironRetrograde after yet another bout of reoccurring auto-immune*”) or Irrelevant (e.g. “*No one is immune to this situation. This is not a chiropractic thing*”), there were 792 remaining tweets that were then stratified by period, Tone and tallied for metrics of engagement (Table 1, Figs. 1, 2 and 3).

**Table 1:** Absolute counts and rates (per month)

Period	Tone	Tweet count	Total likes	Total retweets	Total followers	Count rate	Like rate	Retweet rate	Follower rate
A	Promoting	108	44	23	38,757	36	15	8	12,919
	Refuting	118	1837	558	42,71,250	39	612	186	14,23,750
B	Promoting	194	581	114	12,86,766	16	48	10	1,07,230
	Refuting	180	7053	1484	9,99,842	15	588	124	83,320
C	Promoting	79	61	26	82,939	7	5	2	6912
	Refuting	113	2657	484	4,40,109	9	221	40	36,676

Table 1 and Fig. 1 show that monthly tweet rates declined during the study from a high in period A (36/month promoting, 39/month refuting) to approximately half that in period B (16/month promoting, 15/month refuting) and then approximately half that again in period C (7/month promoting, 9/month refuting).

The ratio of tweets that promoted and refuted a relation between chiropractic/SMT and immunity was approximately equal in all time periods (Table 1, Fig. 2).

Likes, retweets and followers were plotted over time (Fig. 1) with proportions plotted in Fig. 3. Over the study, a much greater proportion of likes were expressed for refuting tweets which captured at least 90% of all likes compared to around 10% for promoting tweets. For Retweets, the proportion in support of refuting tweets remained above 93% for all time periods. In periods A and C, followers of refuting tweets were in the majority at 99% and 84%, respectively. In period B, the percentage of followers between refuting and promoting tweets was roughly equal.

**Fig. 4:**
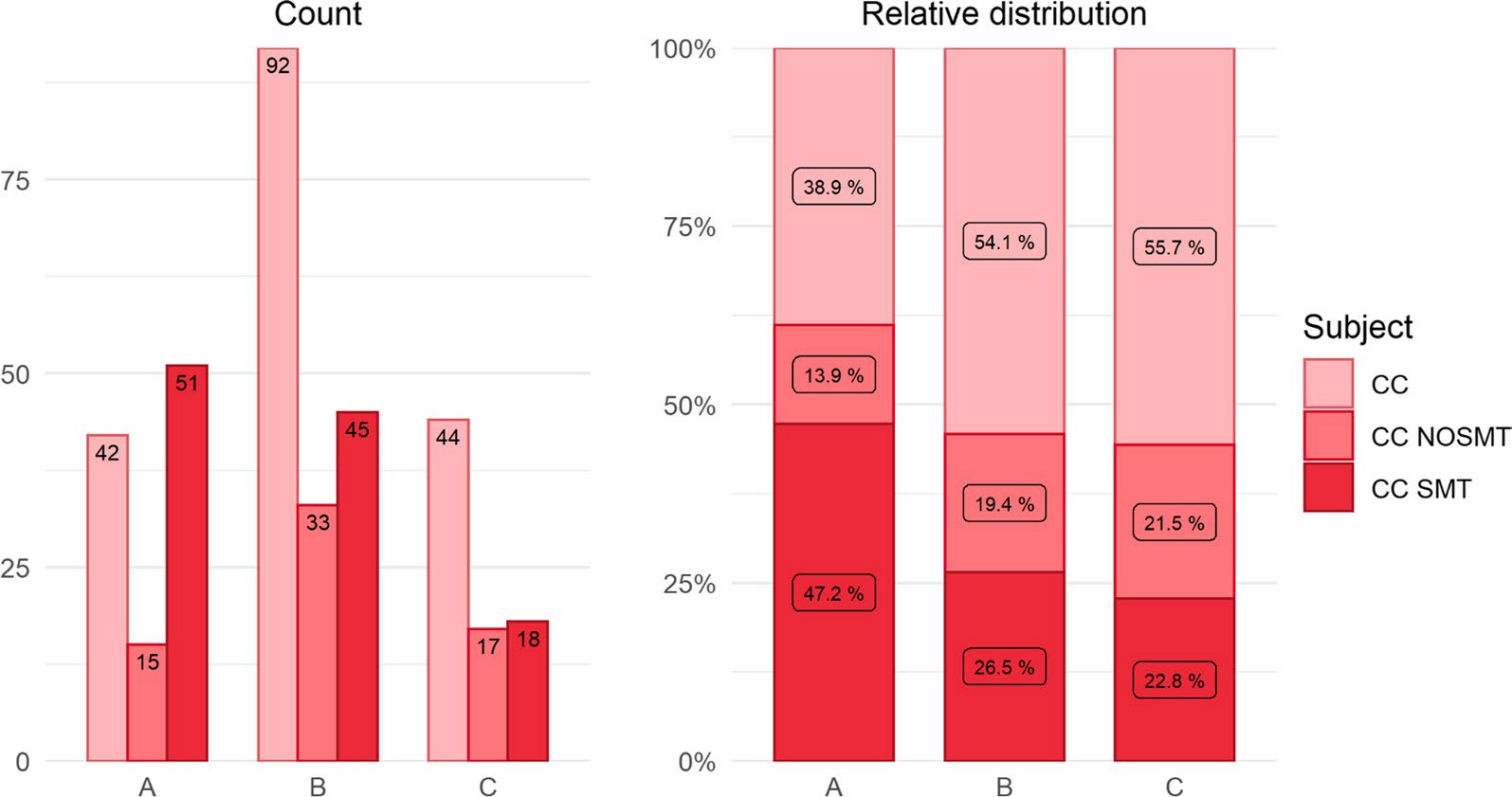
Proportion of subject content (chiropractic/interventions) in promoting tweets (CC, SMT, CC noSMT) in monthly tweet rates for each time period

For tweets promoting a relation between chiropractic/SMT and immunity, tweet content (chiropractic/interventions) for period A (Table 3, Fig. 4) was distributed as follows: CC (38.9%), noSMT (13.9%) and SMT (47.2%). To maintain the same percentage distribution from period A to B, we would expect each of these three content codes to decrease equally by 39% each (Table 3). This was not observed. The proportion of CC, SMT and noSMT tweets changed at different rates from period A to B. Tweets promoting a positive relation between SMT and immunity decreased at a rate of 22% which was 17% faster than expected. Content coded as CC and noSMT decreased to 55% and 55% respectively, which was 15% and 16% slower than expected. Similarly, for periods B–C, the expected rate of decline was 46%. In this period, promoting tweets coded as SMT declined faster than expected (40%) while CC and no SMT tweets declined less quickly than expected (48% and 52% respectively).

**Fig. 5:**
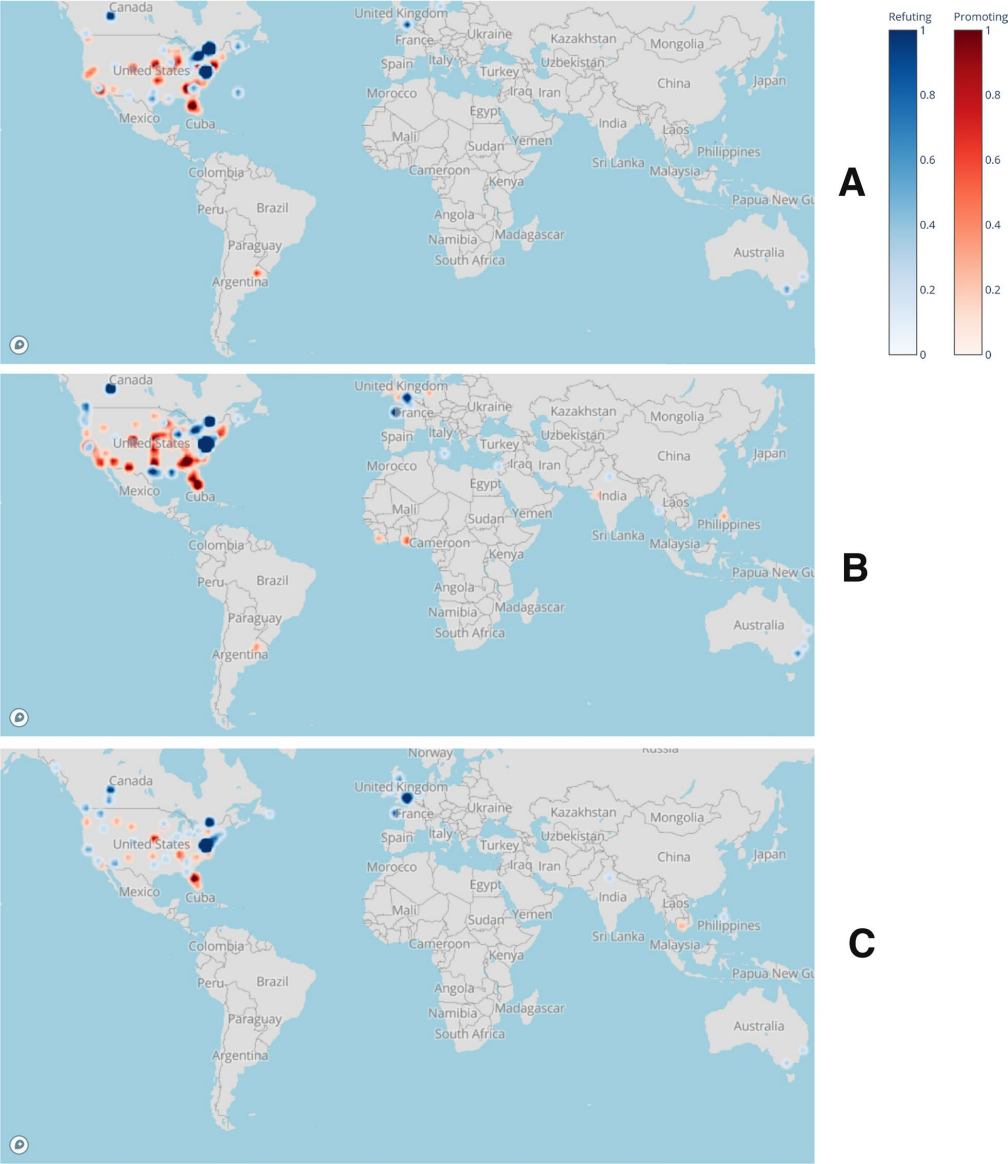
Geospatial heat map of promoting and refuting tweets in time periods A, B and C

Geospatial analysis demonstrated that over all time periods, tweets that promoted a positive relation between chiropractic/SMT and improved immunity originated overwhelmingly in the USA whereas tweets that refuted such a relationship originated primarily in Canada, Europe and Australia. Figure 5 shows the distribution of promoting and refuting tweets for each time period.

**Fig. 6:**
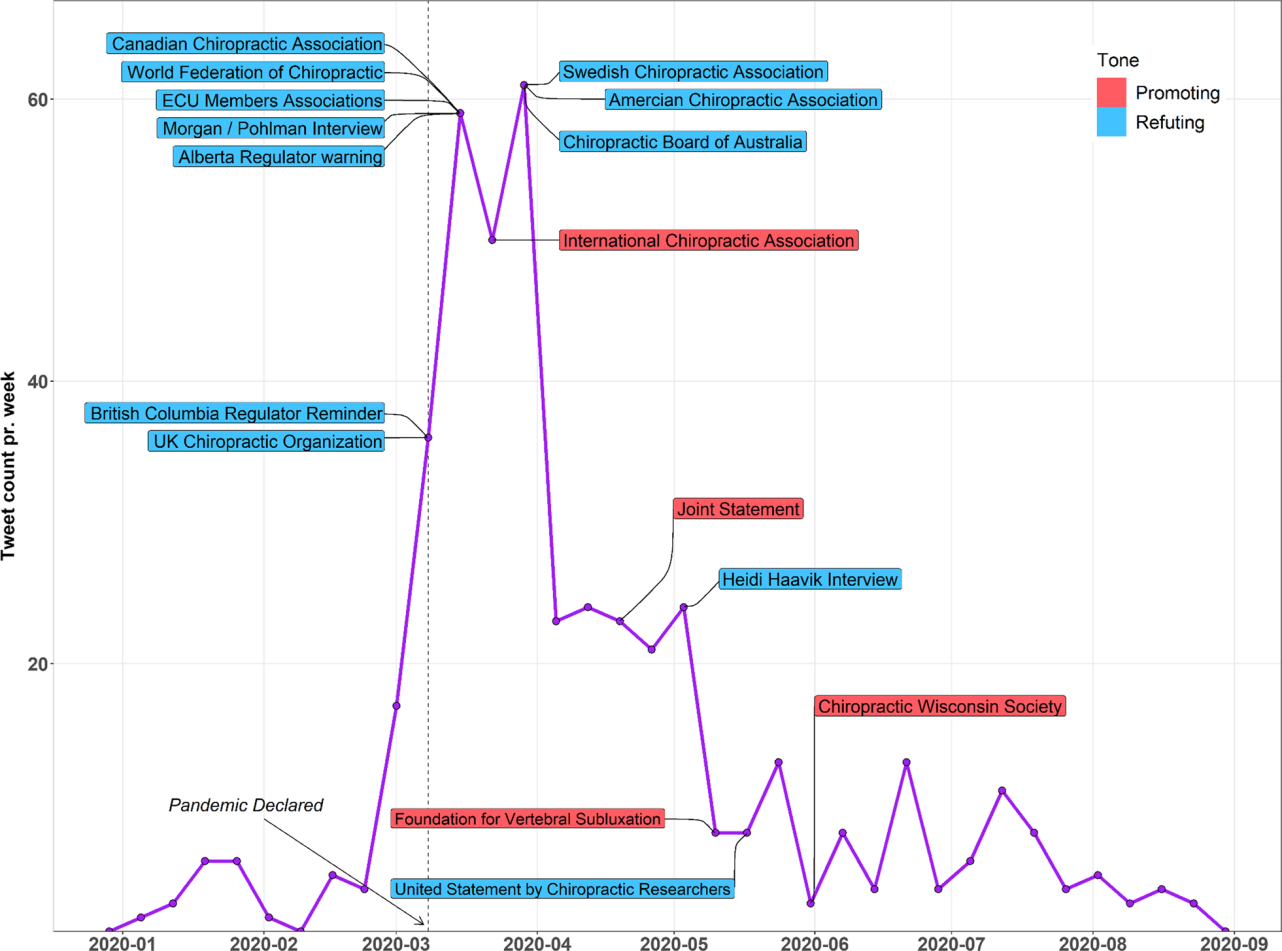
Tweets superimposed with dates of promoting and refuting announcements from the chiropractic profession

Figure 6 plots total Twitter activity over time with the superimposed dates of announcements from chiropractic organizations and regulators that were designed to either refute, or promote, a relation between chiropractic/SMT and immunity.

## Discussion

This paper presents a 27 month time-series analysis of Twitter messaging related to chiropractic/SMT and immunity. From January 1, 2020, Twitter activity increased until March 31, 2020 when it peaked then declined steadily over the remaining 24 months. The ratio of tweets promoting a relation between chiropractic/SMT and immunity and those tweets refuting the same relation, remained relatively constant over the 27 months. Metrics of engagement overwhelmingly supported tweets that refuted a relation between chiropractic/SMT and immunity. Following peak Twitter activity, tweets promoting a relation between chiropractic/SMT and immunity decreased at a rate that was higher than expected. Possible reasons for this observation include efforts by chiropractic organizations and regulators to address misinformation early in the pandemic. There remains a divide between the geospatial origin of tweets promoting a relation between chiropractic/SMT and immunity (United States of America) and tweets refuting this relation (Canada, Europe and Australia).

It should be noted that in our previous paper, chiropractic was mentioned most often in tweets associated with immunity (21%) followed by naturopathy (6%). As a result, this two-year follow-up was limited specifically to the chiropractic profession as it was clearly most often associated with SMT and immunity.

In this two-year follow-up study, our first hypothesis was supported; Twitter activity reached its peak just 20 days following the pandemic declaration. Once reaching its peak, Twitter activity declined steadily without any sign of rebound (Fig. 1, Table 1).

Our second hypothesis was also supported. Through the 27 months of data collection, the ratio of promoting versus refuting tweets remained constant at ~ 50% (Table 2, Fig. 2). This constant ratio suggests that authors of promoting or refuting tweets tend to counter-post in response to tweets of opposing viewpoints thereby balancing out the ratio over time.

**Table 2:** Engagement of tweets (absolute counts and monthly percentages) stratified by period and content

Period	Content	Tweet count	Total likes	Total retweets	Total followers	% Count	% Likes	% Retweets	% Followers
A	CC	42	14	5	17,856	38.9	32	22	46
	CC NOSMT	15	16	14	7329	13.9	36	61	19
	CC SMT	51	14	4	13,572	47.2	32	17	35
B	CC	92	212	49	1,40,176	54.1	37	44	11
	CC NOSMT	33	82	29	5,78,450	19.4	14	26	47
	CC SMT	45	278	33	5,16,349	26.5	49	30	42
C	CC	44	21	7	69,057	55.7	34	27	83
	CC NOSMT	17	37	18	11,787	21.5	61	69	14
	CC SMT	18	3	1	2095	22.8	5	4	3

Interestingly, engagement and reach of promoting versus refuting tweets were far from equivalent. As was the case in our first paper [8], the total likes, retweets and followers of refuting tweets were orders of magnitude greater compared to promoting tweets. The result was that refuting tweets were much more impactful (Table 1, Fig. 3).

While the decline of Twitter activity after March 31, 2020 could be explained by social media fatigue, confusion, or dilution of attention by other media sources [28], we also observed a parallel decrease in tweets with messaging that SMT boosts immunity (Tables 2 and 3. Figure 4). Interestingly, these tweets declined at a higher-than-expected rate (Table 3) compared to tweets suggesting that chiropractic care or nonSMT interventions improve immunity (both of which declined at a less-than-expected rate).

**Table 3:** Overall tweet rates with the expected and actual tweet rates between time periods

										Period A–B			Period B–C		
Content	Number of promoting Tweets			Tweets/m			Proportion of tweets/m (%)			Expected rate of to preserve proportion (%)	Actual rate of change (%)	Difference (%)	Expected rate of to preserve proportion (%)	Actual rate of change (%)	Difference (%)
	Period A	Period B	Period C	Period A	Period B	Period C	Period A	Period B	Period C						
CC	42	92	44	14.00	7.67	3.67	39	54	56	39	55	15	46	48	1
noSMT	15	33	17	5.00	2.75	1.42	14	19	22	39	55	16	46	52	5
SMT	51	45	18	17.00	3.75	1.50	47	26	23	39	**22**	**− 17**	46	**40**	**− 6**
Total	108	170	79	36.00	14.17	6.58	100	100	100						

Bold values indicate a decline in expected tweet rates

The decrease in Twitter activity following March 31, 2020, combined with a coincident decrease in controversial tweets with messaging that SMT boosts immunity, strongly suggests the appearance of some external factor driving these parallel changes. While we cannot confirm the chiropractic announcements plotted in Fig. 6 caused a parallel decline in Twitter activity and SMT messaging, the intended effect was observed; there is quantitatively less misinformation on Twitter regarding SMT and immunity. Interestingly, the timing of these contrary efforts was not associated with any contrary rise in Twitter activity nor contrary increase in SMT messaging.

The resulting decrease in Twitter activity, together with the parallel decrease in tweets linking SMT to improved immunity, may have been sustained by other activities occurring weeks or months after peak Twitter activity and include:

- A unified statement from more than 150 chiropractic researchers against the claim that chiropractic care boosts immunity [29].
- An interview with a prominent chiropractic vitalistic researcher who stated that “because we have no studies yet that look at would chiropractic care prevent you from getting sick or would chiropractic care reduce the symptoms of being sick or the frequency of getting sick? Those studies haven’t been done yet.” [30].
- The emergence of interventions over the course of the pandemic (social distancing, vaccines and anti-viral medications) that mitigated infection and/or serious consequences of covid infection (hospitalization, long-covid, death), acting to make messaging about boosting immunity less relevant, urgent or attention-grabbing.
- Changes in Twitter policy designed to target misinformation, and account owners who distribute misinformation.

Although the overall decline in tweets promoting SMT as a positive influence on immunity is a  desirable development, we note that the remaining proportion of tweets extolling a positive benefit of chiropractic care on immunity is no less of a concern. Although we cannot know the intent of those posting to social media, we suspect that given the factors listed above (especially increased regulatory oversight), some tweet authors may have consciously or unconsciously developed a Trojan Horse strategy by de-emphasizing controversial messaging about SMT while alternatively promoting the profession that provides it. It must be emphasized here that replacing SMT with chiropractic care to suggest a positive effect on immunity, is also misinformation. As is the case with SMT, there is no evidence that chiropractic care, however it may be defined, generates a clinically meaningful improvement in human immunity compared to those withheld from the same intervention. Importantly, we acknowledge studies that report changes in immune parameters following SMT, but these studies have not shown clinical significance in humans. They join an almost endless list of other studies showing any number of changes in anatomy, physiology, various biomarkers and neurology post-SMT. The critical point in the evolution of this body of literature is that for any of these observed changes to be meaningful, these changes must result in a clinically important improvement in human health compared to persons who do not receive the same intervention(s) [11].

Our observation that the majority of promoting tweets originate in the United States is in agreement with the data from our prior paper. While it is difficult to know the global extent of all prompting and refuting messaging outside of Twitter, we also note that announcements from chiropractic organizations that promoted the idea of chiropractic/SMT improving immunity also came primarily from the United States. Explanations for this geographic separation are not readily available, but possible avenues of future investigation may include comparing the proportion of senior versus early career chiropractors in various countries and the location of chiropractic schools that emphasize conservative or dogmatic chiropractic views [31].

### Limitations

It is important to emphasize that it is not possible to confirm the occupation or affiliation of those who author tweets. In addition, Twitter was selected for this study as its entire corpus is searchable. While there is evidence that other social media outlets such as Facebook have many more posts regarding this issue, the majority of these posts occur within private groups and are therefore inaccessible to systematic searching and analysis. The ability to search and track content of Tweets is an advantage and is indicative of the volume of activity in a specific topic. It is also a common way of measuring impact of social media and it is done in many different ways including through Altmetric (www.altmetric.com). However, it must be remembered that measures of engagement do not guarantee that tweets, like any other written content, influences actions or public opinion.

In rating Tweets, the TTI is a new tool that is not used widely, therefore, we took great care discussing its development in our prior paper and then how it was applied here by the same investigators using the same processes to resolve any disagreement.

Finally, it is important to note that we do not infer causal relationships between events and twitter claims; we simply describe what was observed and suggest there is a striking pattern between these events.

## Conclusion

Overwhelmingly, Twitter activity during the COVID-19 pandemic focussed on refuting a relation between chiropractic/SMT and immunity. We observed that a decline in Twitter activity promoting a relation between SMT and immunity coincided with initiatives from chiropractic organizations and regulators to refute these claims. The majority of misinformation about this topic is generated in the United States.

## Data Availability

All data generated or analysed during this study are included in this published article.

## Abbreviations

CC: Chiropractic care
noSMT: Chiropractic care with non-SMT intervention
SMT: Spinal manipulative therapy
TTI: Twitter tone index
